# Logic circuits composed of flexible carbon nanotube thin-film transistor and ultra-thin polymer gate dielectric

**DOI:** 10.1038/srep26121

**Published:** 2016-05-17

**Authors:** Dongil Lee, Jinsu Yoon, Juhee Lee, Byung-Hyun Lee, Myeong-Lok Seol, Hagyoul Bae, Seung-Bae Jeon, Hyejeong Seong, Sung Gap Im, Sung-Jin Choi, Yang-Kyu Choi

**Affiliations:** 1 School of Electrical Engineering, Korea Advanced Institute of Science and Technology, (KAIST) 291 Daehak-ro, Yuseong-gu, Daejeon, 34141, South Korea; 2 School of Electrical Engineering, Kookmin University, Jeongneung-dong, Seongbuk-gu, Seoul, 02707, Republic of Korea; 3 Department of Chemical and Biomolecular Engineering, Korea Advanced Institute of Science and Technology (KAIST), 291 Daehak-ro, Yuseong-gu, Daejeon 34141, South Korea; 4 Graphene Research Center, KI for Nanocentury, KAIST, Daejeon, 34141, South Korea

## Abstract

Printing electronics has become increasingly prominent in the field of electronic engineering because this method is highly efficient at producing flexible, low-cost and large-scale thin-film transistors. However, TFTs are typically constructed with rigid insulating layers consisting of oxides and nitrides that are brittle and require high processing temperatures, which can cause a number of problems when used in printed flexible TFTs. In this study, we address these issues and demonstrate a method of producing inkjet-printed TFTs that include an ultra-thin polymeric dielectric layer produced by initiated chemical vapor deposition (iCVD) at room temperature and highly purified 99.9% semiconducting carbon nanotubes. Our integrated approach enables the production of flexible logic circuits consisting of CNT-TFTs on a polyethersulfone (PES) substrate that have a high mobility (up to 9.76 cm^2^ V^−1^ sec^−^1), a low operating voltage (less than 4 V), a high current on/off ratio (3 × 10^4^), and a total device yield of 90%. Thus, it should be emphasized that this study delineates a guideline for the feasibility of producing flexible CNT-TFT logic circuits with high performance based on a low-cost and simple fabrication process.

Printable flexible electronics is considered as one of the most rapidly developing technologies because it has the potential to be scalable and cost-effective^1^,^2^,^3^,^4^,^5^. In particular, printed thin-film transistors (TFTs) that utilize carbon nanotubes (CNTs) have exhibited high on-state currents, mobility, and current on/off ratios at low operating voltages. These CNT-TFTs have been fabricated by various printing techniques, such as roll-to-roll gravure^6^,^7^, aerosol jet^8^,^9^,^10^, and screen printing^11^. However, the roll-to-roll gravure and aerosol jet printing processes present challenges because the high roughness of the resulting layers limits the precision in patterning electrodes^6^,^7^. In addition, the reported screen printing process presented inherent issues, such as a thick printed layer, because it requires ink with high viscosity^11^. Therefore, the reported CNT-TFTs produced using the aforementioned methods resulted in low carrier mobility and correspondingly high operating voltages^6^,^7^,^11^.

On the other hand, the inkjet printing technique may be the most promising alternative because of its mask-less process, high printing resolution, and low-temperature requirement^12^,^13^,^14^,^15^,^16^,^17^,^18^,^19^. Therefore, a number of studies on the inkjet-printed CNT-TFTs based on various gate dielectric layers, such as ion-gel materials^9^,^12^,^13^, barium titanate (BaTiO3-BTO) nanoparticles^6^,^11^,^14^, and polymethyl methacrylate (PMMA)^15^, have been extensively reported. However, these dielectric layers have several additional constraints, including an increased thickness, low dielectric constant, pinholes, and solvent residues, which inevitably necessitate high operation voltage and deteriorate device yield.

To address the aforementioned concerns, we previously reported the preparation of poly(1,3,5-trimethyl-1,3,5-trivinyl cyclotrisiloxane) (pV3D3) via a one-step, solvent-free technique called ‘initiated chemical vapor deposition’ (iCVD), which produces a versatile polymeric insulating layer that meets a wide range of requirements for next-generation ‘soft’ electronic devices^20^,^21^,^22^. In this work, we demonstrate that the iCVD pV3D3 layer can be used as a gate insulator for high-performance inkjet-printed top-gate CNT-TFTs that are implemented on a flexible polymeric (polyethersulfone, PES) substrate. We used highly purified semiconducting (99.9%) CNTs to reinforce the device yield and improve the electrical performance of the devices^23^. Our results show that the iCVD process enables the formation of a highly uniform and ultrathin film of pV3D3 that has excellent insulating properties^20^. The fabricated inkjet-printed CNT-TFTs with the pV3D3 gate insulating layer showed excellent electrical properties in terms of mobility (up to 9.76 cm^2^/V·sec), the on/off current ratio (3 × 10^4^), the operation voltage (<4 V), and device yield (close to 90%). In addition, logic circuits consisting of the inkjet-printed top-gate CNT-TFTs, including the inverter, NAND, and NOR, were fabricated. The proposed platform of the printed CNT-TFTs with a polymeric gate insulator has the potential to serve as a foundation for scalable, low-cost, high-performance flexible and large-scale future soft electronics.

## Results and Discussion

**Figure 1:**
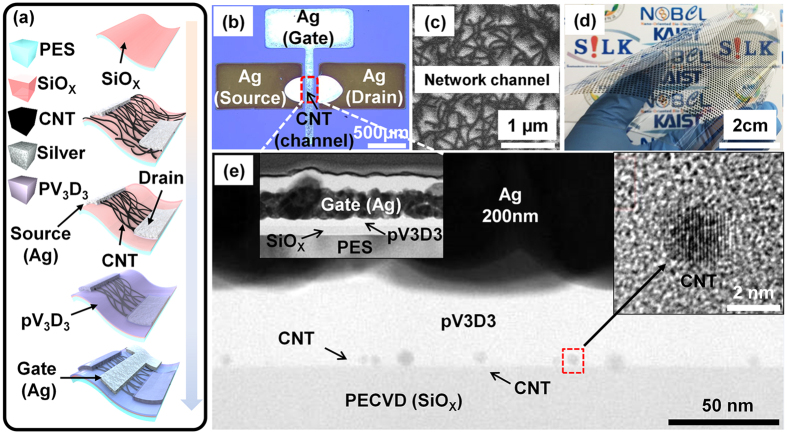
Device schematic and various device images of the printed top-gate CNT-TFTs using 99.9% semiconducting nanotubes. (**a**) Schematic diagram showing the printing process scheme. (**b**) Representative optical image of a CNT network deposited on a substrate. (**c**) SEM image of the PES surface after CNT deposition. (**d**) Photograph of the final 44 × 40 device array on a PES substrate. (**e**) Cross-sectional TEM image of the printed top-gate CNT-TFTs.

Figure 1(a) depicts the fabrication process of the flexible inkjet-printed CNT-TFTs with a polymeric gate insulator. The substrate was then functionalized to introduce an amine-terminated adhesion layer for the deposition of semiconducting CNTs. After that, to deposit a random network of CNTs, the substrate was then immersed in a high-purity 99.9% semiconducting SWNT solution^23^,^24^. Subsequently, To form source/drain electrodes, a conductive nanoparticle silver (Ag) ink was deposited by an inkjet printer^25^. Then, the channel area was defined by an additional PVP printing step, followed by O_2_ plasma etching to etch away the unwanted CNTs^24^. To form the gate insulator on the device, a pV3D3 formed via the iCVD process (refer to Supplementary Information S1). In the end, Ag gate was printed by the same method as the source/drain printing. Figure 1(b) depicts an optical image of the printed top-gate CNT-TFT. The length (*L*) and the width (*W*) of the CNT network channel (red dashed line) were approximately 350 ± 25 μm and 200 ± 25 μm, respectively. Figure 1(c) illustrates a scanning electron microscope (SEM) image of the CNT network channel. The SEM image revealed that the semiconducting CNTs deposited from the solution were uniformly distributed on the PECVD SiO_x_ surface; this uniformity is crucial for achieving uniform device properties and logic gate performance. The average CNT density obtained with a deposition time of 7 hr was approximately 10 tubes/μm. Figure 1(d) shows a 44 × 40 array of top-gate CNT-TFTs printed on the flexible PES substrate. The cross-sectional transmission electron microscopy (TEM) image in Fig. 1(e) shows that the 40 nm thick pV3D3 dielectric was deposited with an excellent uniformity on the large-scale area by using the initiated chemical vapor deposition (iCVD). In order to show the scalability of the pV3D3 film thickness, a simple capacitor composed of Al/pV3D3/Al metal/insulator/metal (MIM) devices was fabricated and electrical characteristics were included in the Supplementary Information S2. Note that the optimal substrate temperature was near room temperature for the iCVD-grown pV3D3 layer, which enables the non-destructive deposition of pV3D3 onto an underlying substrate that is thermally and chemically sensitive^20^,^22^. The components of the fabricated device, such as the gate electrode (Ag), the dielectric layer (pV3D3), the CNT network channel, and the substrate (SiO_X_) were confirmed by X-ray photoelectron spectroscopy (XPS) and energy-dispersive X-ray spectroscopy (EDS) mapping analyses (refer to Supplementary Information S3). Impurities were not observed because of the inherent solvent-free process during the iCVD. The inset in Fig. 1(e) is a cross-sectional TEM image that shows that the thicknesses of the printed gate electrode, the gate insulator, and the SiO_X_ substrate were approximately 200 nm, 40 nm, and 50 nm, respectively.

**Figure 2:**
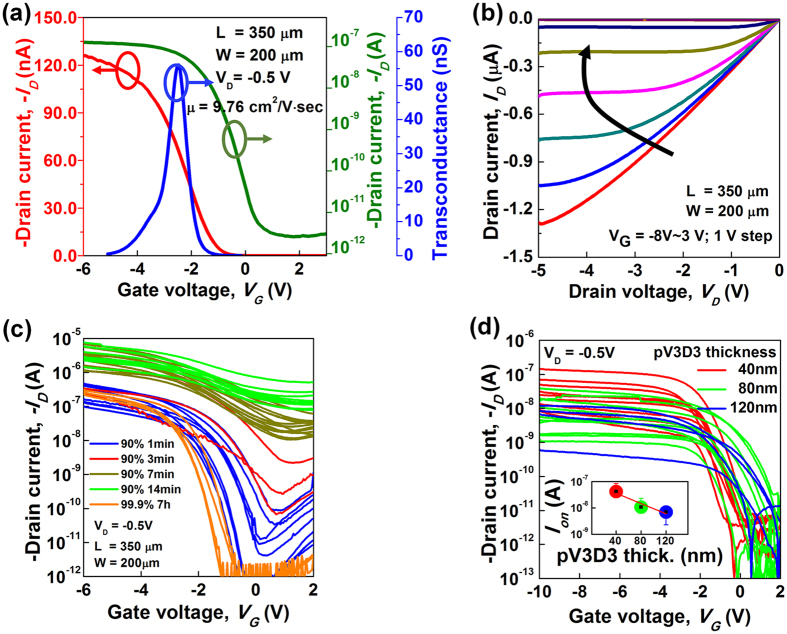
(**a**) Transfer characteristics (green: log scale, red: linear scale) and *g*_m_ vs. *V*_G_ (blue) of the printed top-gate CNT-TFTs (*L* = 350 μm and *W* = 200 μm). (**b**) Output characteristics with various *V*_*G*_ values ranging from −8 to 3 V. (**c**) Transfer characteristics with two semiconducting enriched nanotube solutions (90% and 99.9%) and various deposition times. (**d**) Transfer characteristics for several pV3D3 gate dielectric thicknesses (inset). The values of *I*_*ON*_ value were extracted from the transfer characteristics for each condition.

The electrical characteristics of the printed top-gate CNT-TFTs on the PES substrate were measured at room temperature. High-performance CNT-TFT operations with low gate leakage currents were achieved with various thicknesses of pV3D3 (*t*_*pV3D3*_) ranging from 40 to 120 nm. The representative transfer characteristics with transconductance (*g*_*m*_) are presented in Fig. 2(a). The fabricated CNT-TFTs normally exhibited p-type behavior in the ambient environment because of oxygen and moisture adsorption. The measured off- and on-state currents at *V*_*D*_ −0.5 V were 2.5 pA, and 132 nA, respectively, the peak value of *g*_*m*_ was 56.1 nS, and the corresponding current on/off ratio remained above 10^4^ on average. It is believed that this level of performance is sufficient for wide logic gate applications. The charge carrier mobility value was also extracted based on the *g*_*m*_. At *V*_*D*_ −0.5 V, the device operates in the linear regime; therefore, the carrier mobility could be calculated from the following equation


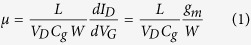


where *C*_*g*_ is the gate capacitance per unit area. The value of *C*_*g*_ was calculated through the well-known cylindrical model by considering the electrostatic coupling and the quantum capacitance of the CNTs as follows:


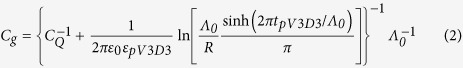


where *Λ*_*0*_^−1^ denotes the density of the CNTs (in our case, 10 tubes/μm), *C*_*Q*_ is the quantum capacitance (4.0 × 10^−10^ F/m), and *R* is the average radius of the CNTs (0.6 nm)^15^,^16^,^17^. In addition, the permittivity of pV3D3 (*ε*_*pV3D3*_) is 2.2 *ε*_*o*_ where *ε*_*o*_ is the permittivity of a vacuum^20^ and *t*_*pV3D3*_ is 40 nm. In this study, the value of *C*_*g*_ was determined to be 2.267 × 10^−10^ F/cm^2^. Based on the device geometry, the transconductance normalized by the channel width (i.e., *g*_*m*_/*W*) and *C*_*g*_, carrier mobility was calculated to be 9.76 cm^2^/V·sec for the extracted from the fabricated inkjet-printed CNT-TFTs.

Figure 2(b) presents the output transfer characteristics (*I*_*D*_ − *V*_*D*_) of the same device at various values of *V*_*G*_ ranging from −8 to 3 V. They follow behaviors of a typical conventional field-effect transistor. Under a small *V*_*D*_ bias regime, the output characteristics appeared to be almost linear, which indicated that there was a low electronic barrier between the Ag S and D electrodes and the semiconducting CNTs.

To gain a more comprehensive understanding of the electrical performance of the devices, CNT-TFTs were produced from 90% semiconducting CNT solution for diverse deposition times was compared with that of devices produced from 99.9% semiconducting CNT solution, with the results shown in Fig. 2(c). In this analysis, we fabricated inkjet-printed top-gate CNT-TFTs with the pV3D3 polymer dielectric on a rigid silicon dioxide surface rather than a flexible PES substrate for simplicity. It can be observed that as the deposition time is increased from 1 to 14 min to form a high-density CNT network from the 90% semiconducting CNT solution, the average on-state current is increased from 0.27 μA to 3.43 μA. However, the on/off current ratio rapidly decreases because the probability of a metallic interconnections between the source and drain electrodes increases with the CNT density^23^,^24^. Although the electrical performances, including the on-state current and on/off current ratio, were similar between the devices fabricated from 90% semiconducting CNTs with a 1-min deposition time and the devices fabricated from 99.9% semiconducting CNTs with a 7-hr deposition time, the degree of device uniformity was significantly different. It was also observed that fluctuations of the on-state current were smaller in the devices with 99.9% semiconducting CNTs than those of 90% semiconducting CNTs, as expected. In general, the statistical nature of the CNT-TFTs causes a number of expected variations in the electrical performances because of variations in the number of connecting paths and the portion of metallic CNTs between the source and drain electrodes. Therefore, the devices with lower-density networks (i.e., 90% at 1 min) were generally more affected by these variations than those with higher-density networks (i.e., 99.9% at 7 hr). Thus, in the devices with the higher-density networks, a reduction in variations was expected because of the averaging effect^26^. Therefore the CNT-TFTs comprised of the highly purified semiconducting nanotubes (99.9%) with a longer deposition time was fabricated to improve device uniformity. Moreover, the off-state current of the CNT-TFTs with 99.9% CNTs was apparently smaller than that with 90% CNTs. Hence lowered static power consumption is expected.

We also investigated thickness controllability of the pV3D3 polymer dielectric, which would improve the electrical performance of the CNT-TFTs as shown in Fig. 2(d). In detail, the accurate thickness controllability of the iCVD permits the use of an ultrathin polymer gate dielectric layer that is much thinner than that other gate dielectrics used in printable electronics such as ion-gel, BaTiO_3_-BTO, and PMMA. Their thicknesses are in a range of 0.5 ~ 5 μm^6^,^7^,^11^,^13^,^14^. The ultrathin gate dielectric induces strong gate capacitance, and the thinner layer results in an increase of the on-state current under the same gate bias. Compared to previous works^6^,^7^,^11^,^14^, our results represent the improvement of the on/off ratio and channel mobility under the low operating voltage (>4 V).

In addition, critical drawbacks are not observed when using this engineering approach. The insulating properties and the thickness scalability of the pV3D3 are better than organic gate dielectric layers (refer to Supplementary Information S4)^20^,^22^.

**Figure 3:**
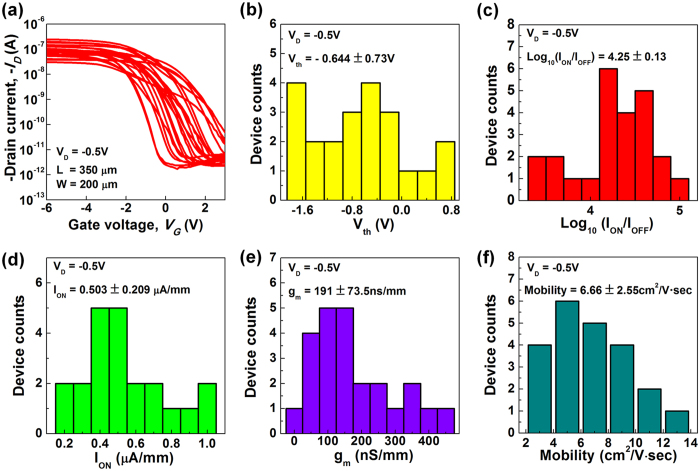
Distributions of the electrical properties of the printed top-gate CNT-TFT arrays. (**a**) Transfer characteristics of 23 CNT-TFTs measured at *V*_*D*_ − 0.5 V. (**b**–**f**) Histograms of *V*_*th*_, *I*_*ON*_*/I*_*OFF*_, *I*_*ON*_, transconductance (*g*_*m*_), and field-effect mobility.

Next, we focused on the uniformity of the fabricated devices as shown in Fig. 3. The functional device yield was close to 90%, and the rest of the devices had fractures in the electrodes because of manual handling of the devices. These failures occurred because of weak adhesion between the Ag nanoparticles and the substrate and because of the rough surfaces of the electrodes. Figure 3(a) shows the transfer characteristics of 23 CNT-TFTs (L = 350 μm and W = 200 μm) measured at *V*_*D*_ of −0.5 V. And Fig 3(b–f) show the histograms of the statistical variations of the threshold voltage (*V*_*th*_), *I*_*ON*_/*I*_*OFF*_, *I*_*ON*_/*W*, *g*_*m*_/*W*, and mobility, respectively. *V*_*th*_ was extracted in the linear region of the *I*_*D*_ − *V*_*G*_ characteristics at *V*_*D*_ of −0.5 V. The best measured performances were *I*_*ON*_/*I*_*OFF*_ of 5.2 × 10^4^, *I*_*ON*_/*W* of 1.26 μA/mm, *g*_*m*_/*W* of 418 nS/mm, and mobility were 14.56 cm^2^/V·sec. The average values of *I*_*ON*_/*I*_*OFF*_, *I*_*ON*_/*W*, *g*_*m*_/*W*, and mobility are 4.25 ± 0.13, 0.5 ± 0.2 μA/mm, 191 nS/mm, and 6.66 ± 2.55 cm^2^/(V·sec), respectively. Further improvements in the yield and the consistency uniformity may be possible through refinements to the process.

To determine the suitability of the fabricated top-gate CNT-TFTs with the pV3D3 gate dielectric for flexible electronic devices, the mechanical flexibility was evaluated. The proposed CNT-TFT is inherently advantageous with regards to mechanical flexibility as long as the printed metal and the dielectric layer remain stable and/or flexible as a result of the small diameter of the CNTs. Importantly, the pV3D3 gate dielectric has excellent mechanical flexibility because of the elastic hexagonal siloxane structure of the pV3D3 layer^20^. (refer to Supplementary Information S5).

**Figure 4:**
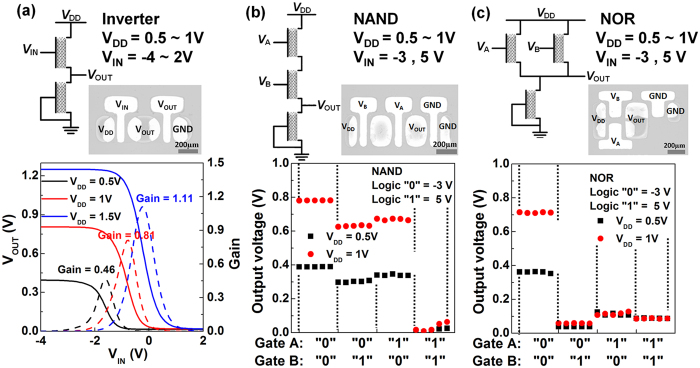
Inverter, 2-input NAND and NOR circuits using all p-type printed top-gate CNT-TFTs on a PES substrate with *V*_*DD*_ 0.5 V, 1 V and 1.5 V. (**a**) Schematic of the diode-load inverter using the CNT-TFTs with all p-type devices, an optical microscope image, and the inverter voltage transfer characteristic (solid lines) and the voltage gain (dashed lines). (**b**,**c**) Output voltages of the 2-input NAND and NOR circuit, respectively, where supply voltages *V*_*DD*_ of 0.5 V and 1 V were applied. The input voltage of −3 V and 5 V are treated as logical “1” and “0”, respectively.

We designed the printed logic gates, including resistive load p-type only inverters (Fig. 4(a)), 2-input NAND (Fig. 4(b)), and 2-input NOR (Fig. 4(c)) gates, with the printed top-gate CNT-TFTs (*L* = 200 μm and *W* = 350 μm) on a PES substrate. Figure 4(a) describes the circuit diagram of the all p-type diode-load inverter with the input, output, and drain voltage. The inverter exhibited well-defined static voltage transfer characteristics. The output swing was approximately 80% level at a *V*_*DD*_ of 0.5 V, 1 V and 1.5 V, which is comparable to that of previously reported flexible inverters^3^,^15^. A low output swing is a general property in logic gates, especially those consisting of all p-type CNT-TFTs. Hence, *V*_*OUT*_ may not be pulled exactly to *V*_*DD*_ or ground, which indicates the presence of a small static current. Furthermore, the NAND and NOR logic gates were fabricated utilizing the inverter components as shown in Fig. 4(b,c). Both types were evaluated with *V*_*DD*_ 0.5 V and 1 V. Voltages of −3 V and 5 V were applied to Gate A, to produce a logical “1” and “0”, respectively, at Gate B. These output characteristics confirm the feasibility of the fabricated circuits for the realization of logic functions. In addition to higher logic performance, the range of device parameters need to be further tightly controlled to achieve optimum performance. Although many challenges remain, the logic circuits produced with an ultrathin pV3D3 polymer gate dielectric and highly purified semiconducting CNTs may represent an important milestone for consecutive CNT logic technology developments.

In this study, we present the inkjet-printed technique for fabricating flexible top-gate CNT-TFT logic circuits with an ultrathin pV3D3 polymer gate dielectric and highly purified semiconducting CNTs. To the best of our knowledge, this is the first time that the iCVD process and an inkjet printing method have been used to construct an ultrathin polymeric gate dielectric layer for the CNT-TFTs. Incorporating the efficient methods employed in this study provides high-performance characteristics, including flexibility, stable operations, high mobility, a low operation voltage, a high current on/off ratio, and a high yield, based on a low-cost and simple fabrication process. Thus, our integrated approach has potential for use in the production of extremely low-cost and large-scale flexible device.

## Methods

### Fabrication of Flexible CNT-TFT circuits

Flexible CNT-TFTs were fabricated on polyethersulfone (PES, SKC Co.) First, a 50-nm thick layer of SiO_X_ was formed using plasma-enhanced chemical vapor deposition (PECVD) at 150 °C. Then the substrate was first functionalized to form an amine-terminated adhesion layers. (Poly-L-lysine aqueous solution, 0.1% w/v in water; Sigma-Aldrich) Subsequently, the substrate was immersed in highly purified 99.9% semiconducting enriched CNT solution (provided from NanoIntegris, Inc.) for 7 hr to deposit a random network of CNTs, and it was then rinsed with deionized water. Subsequently, a conductive nanoparticle silver (Ag) ink (InkTec Tec-IJ-060) was deposited by an inkjet printer (UJ200MF, Unijet) integrated with a single piezoelectric-type dispenser (MicroFab) with 50-μm orifice nozzles to form the source and drain (S/D) electrodes. Prior to the inkjet printing, the Ag ink was filtered through a 5- μm polytetrafluoroethylene (PTFE) syringe filter to prevent Ag nanoparticle aggregation. The typical inkjet droplet volume ranged from 40 to 50 pL, and the droplet size was approximately 100 μm in diameter. The optimized inkjet printing conditions were drop pitches of 80 to 90 μm and a frequency of 300 Hz. Second, the channel area was defined by an additional printing step using polyvinylpyrrolidone (PVP), which was followed by O_2_ plasma etching to remove the unwanted CNTs outside the channel area. To form the gate insulator on the device, a 40 nm thick layer of pV3D3 was deposited using a custom-made iCVD system. Depositing Poly(1,3,5-trimethyl-1,3,5-trivinyl cyclotrisiloxane) (pV3D3) via the Initiated Chemical Vapor Deposition (iCVD) : 1,3,5-trimethyl-1,3,5-trivinyl cyclotrisiloxane (V3D3, Gelest, 95%) and tert-butyl peroxide (TBPO, 97%, Sigma Aldrich) were employed as the monomer and the initiator, respectively. After vaporization of V3D3 and TBPO, both materials were delivered to a custom-made iCVD reactor. The process pressure was 300 mTorr and the filament was heated to 200 °C. The flow rate of the V3D3 and the TBPO was maintained at 2.5 and 1 sccm by a needle valve. The substrate temperature was maintained at 40 °C for the full process. The deposition rate of the pV3D3 layer was controlled *in situ* by the interferometer. Finally, Ag gate electrodes were printed using the same method employed for S/D printing.

### Characterization

SEM (model S-4800) was employed for visual analysis of the CNT network channel fabricated on PECVD SiO_X_ substrates (Fig. 1c). In order to prepare the sample for TEM, a focused ion beam (FIB, model Helios Nanolab) was employed after coating carbon and platinum for passivation of the sample. The cross-sectional image was obtained from high-resolution TEM (model JEM-ARM200F) (Fig. 1e), and the components of the memory were analyzed by EDS mapping (model Quantax 400) (refer to Supplementary Information S3). All electrical measurements were carried out without any device encapsulation. Electrical measurements were carried out using a HP4156 semiconductor parameter analyzer under ambient conditions. The variations of the capacitance as a function of dielectric thickness were evaluated by use of the simplest MIM structure was characterized with the aid of the LCR meter (HP4284A, Agilent). A custom-made bending machine (refer to Supplementary Information S5) was used to evaluate the flexibility and the bending endurance, respectively.

## Additional Information

**How to cite this article**: Lee, D. *et al.* Logic circuits composed of flexible carbon nanotube thin-film transistor and ultra-thin polymer gate dielectric. *Sci. Rep.* **6**, 26121; doi: 10.1038/srep26121 (2016).

## Supplementary Material

Supplementary Information

## References

[b1] Park S., Vosguerichian M. & Bao Z. A review of fabrication and applications of carbon nanotube film-based flexible electronics. Nanoscale 5, 1727 (2013). 10.1039/c3nr33560g 23381727

[b2] Franklin a. D. Nanomaterials in transistors: From high-performance to thin-film applications. Science 349, aab2750–aab2750 (2015). 26273059 10.1126/science.aab2750

[b3] Peng L., Zhang Z. & Wang S. Carbon nanotube electronics: recent advances. Materials Today 17, 433–442 (2014).

[b4] Wang C., Takei K., Takahashi T. & Javey A. Carbon nanotube electronics – moving forward. Chem. Soc. Rev. 42, 2592–2609 (2013). 23229523 10.1039/c2cs35325c

[b5] Kang S. J. *et al.* High-performance electronics using dense, perfectly aligned arrays of single-walled carbon nanotubes. Nature Nanotechnology 2, 230–236 (2007). 10.1038/nnano.2007.77 18654268

[b6] Lau P. H. *et al.* Fully Printed, High Performance Carbon Nanotube Thin-Film Transistors on Flexible Substrates. Nano Letters 13, 3864–3869 (2013). 23899052 10.1021/nl401934a

[b7] Yeom C. *et al.* Large-Area Compliant Tactile Sensors Using Printed Carbon Nanotube Active-Matrix Backplanes. Advanced Materials 27, 1561–1566 (2015). 25640804 10.1002/adma.201404850

[b8] Ha M. *et al.* Aerosol Jet Printed, Low Voltage, Electrolyte Gated Carbon Nanotube Ring Oscillators with Sub-5 μs Stage Delays. Nano Letters 13, 954–960 (2013). 23394463 10.1021/nl3038773

[b9] Ha M. *et al.* Printed, Sub-3V Digital Circuits on Plastic from Aqueous Carbon Nanotube Inks. ACS Nano 4, 4388–4395 (2010). 20583780 10.1021/nn100966s

[b10] Xu W. *et al.* Flexible logic circuits based on top-gate thin film transistors with printed semiconductor carbon nanotubes and top electrodes. Nanoscale 6, 14891–14897 (2014). 25363072 10.1039/c4nr05471g

[b11] Cao X. *et al.* Screen Printing as a Scalable and Low-Cost Approach for Rigid and Flexible Thin-Film Transistors Using Separated Carbon Nanotubes. ACS Nano 8, 12769–12776 (2014). 25497107 10.1021/nn505979j

[b12] Chen P. *et al.* Fully Printed Separated Carbon Nanotube Thin Film Transistor Circuits and Its Application in Organic Light Emitting Diode Control. Nano Letters 11, 5301–5308 (2011). 22050730 10.1021/nl202765b

[b13] Sajed F. & Rutherglen C. All-printed and transparent single walled carbon nanotube thin film transistor devices. Applied Physics Letters 103, 143303 (2013).

[b14] Cai L., Zhang S., Miao J., Yu Z. & Wang C. Fully Printed Foldable Integrated Logic Gates with Tunable Performance Using Semiconducting Carbon Nanotubes. Advanced Functional Materials 25, 5698–5705 (2015).

[b15] Cao Q. *et al.* Medium-scale carbon nanotube thin-film integrated circuits on flexible plastic substrates. Nature 454, 495–500 (2008). 18650920 10.1038/nature07110

[b16] Shi J., Guo C. X., Chan-Park M. B. & Li C. M. All-Printed Carbon Nanotube finFETs on Plastic Substrates for High-Performance Flexible Electronics. Advanced Materials 24, 358–361 (2012). 22162001 10.1002/adma.201103674

[b17] Kim B. *et al.* High-speed, inkjet-printed carbon nanotube/zinc tin oxide hybrid complementary ring oscillators. Nano Letters 14, 3683–3687 (2014). 24849313 10.1021/nl5016014

[b18] Kim B. *et al.* Low voltage, high performance inkjet printed carbon nanotube transistors with solution processed ZrO2 gate insulator. Applied Physics Letters 103, 082119 (2013).

[b19] Lee C. W. *et al.* High-Performance Inkjet Printed Carbon Nanotube Thin Film Transistors with High-k HfO2 Dielectric on Plastic Substrate. Small 8, 2941–2947 (2012). 22763948 10.1002/smll.201200041

[b20] Moon H. *et al.* Synthesis of ultrathin polymer insulating layers by initiated chemical vapour deposition for low-power soft electronics. Nature Materials 14, 628–635 (2015). 25751074 10.1038/nmat4237

[b21] Petruczok C. D., Chen N. & Gleason K. K. Closed Batch Initiated Chemical Vapor Deposition of Ultrathin, Functional, and Conformal Polymer Films. Langmuir 30, 4830–4837 (2014). 24713046 10.1021/la500543d

[b22] Seong H., Baek J., Pak K. & Im S. G. A Surface Tailoring Method of Ultrathin Polymer Gate Dielectrics for Organic Transistors: Improved Device Performance and the Thermal Stability Thereof. Advanced Functional Materials 25, 4462–4469 (2015).

[b23] Lee D. *et al.* High-performance thin-film transistors produced from highly separated solution-processed carbon nanotubes. Applied Physics Letters 104, 143508 (2014).

[b24] Yoon J. *et al.* Accurate extraction of mobility in carbon nanotube network transistors using C-V and I-V measurements. Applied Physics Letters 105, 212103 (2014).

[b25] Kim K., Ahn S. Il & Choi K. C. Simultaneous synthesis and patterning of graphene electrodes by reactive inkjet printing. Carbon 66, 172–177 (2014).

[b26] Choi S., Bennett P., Lee D. & Bokor J. Highly uniform carbon nanotube nanomesh network transistors. Nano Research 8, 1320–1326 (2015).

