# Supplementary material for: Logic circuits composed of flexible carbon nanotube thin-film transistor and ultra-thin polymer gate dielectric
Source: Sci Rep. 2016 May 17;6:26121. doi: 10.1038/srep26121 (PMC4869014; doi:10.1038/srep26121)
Supplement: Supplementary Information [file srep26121-s1.doc]

**Supporting Information**

**Logic circuits composed of flexible carbon nanotube thin-film transistor and ultra-thin polymer gate dielectric**

*Dongil Lee,^1^ Jinsu Yoon,^2^ Juhee Lee,^2^ Byung-Hyun Lee,^1^ Myeong-Lok Seol,^1^* *Hagyoul Bae,^1^ Seung-Bae Jeon,^1^ Hyejeong Seong,^3,4^ Sung Gap Im,^3,4^ Sung-Jin Choi^2,a)^ and Yang-Kyu Choi,^1,a)^*

^1^School of Electrical Engineering, Korea Advanced Institute of Science and Technology, (KAIST) 291 Daehak-ro, Yuseong-gu, Daejeon, 34141, South Korea

^2^School of Electrical Engineering, Kookmin University, Jeongneung-dong, Seongbuk-gu, Seoul, 02707, Republic of Korea

^3^Department of Chemical and Biomolecular Engineering, Korea Advanced Institute of Science and Technology (KAIST), 291 Daehak-ro, Yuseong-gu, Daejeon 34141, South Korea

^4^Graphene Research Center, KI for Nanocentury, KAIST, Daejeon, 34141, South Korea

1. Authors to whom correspondence should be addressed.

Email addresses: sjchoiee@kookmin.ac.kr and [ykchoi@ee.kaist.ac.kr](mailto:ykchoi@ee.kaist.ac.kr)

**Initiated chemical vapor deposition (iCVD) polymerization**

Figure S1 shows an illustration of the iCVD polymerization process. Using the iCVD process, a monomer (1,3,5-trimethyl-1,3,5-trivinyl cyclotrisiloxane) (V3D3) was polymerized to produce pV3D3 for the polymeric gate dielectrics. The use of the iCVD process for creating the pV3D3 gate dielectric has the advantages of high purity, a low process temperature, and conformal surface growth.


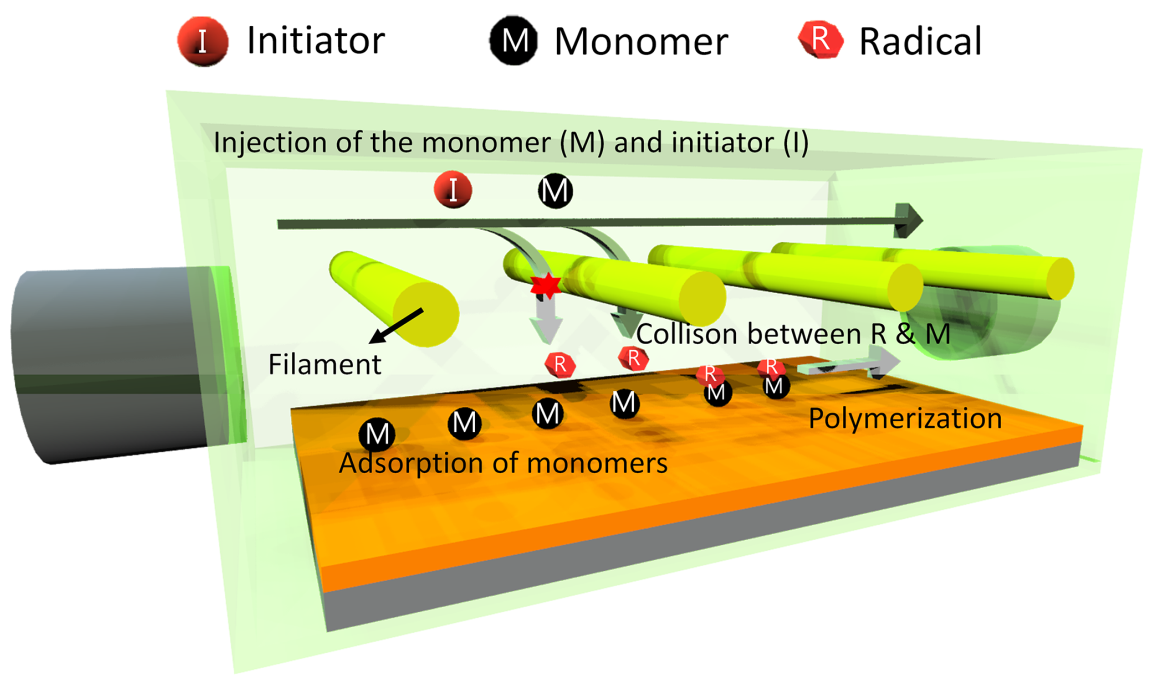


**Figure S1.** Illustration of the iCVD polymerization process.

**References**

1. Coclite, A. M. *et al*. CVD Polymers: A New Paradigm for Surface Modification and Device Fabrication. *Adv. Mater.* **25**, 5392—5423 (2013).
2. Asatekin, A. *et al*. Designing polymer surfaces via vapor deposition. *Mater. Today.* **13**, 26—33 (2010).

**Capacitance versus thickness of the pV3D3 dielectric layer is analyzed by use of the simple Al/pV3D3/Al capacitor**

Figure S2 presents variation of capacitance as a function of pV3D3 dielectric thickness for the Al/pV3D3/Al MIM devices. It is found that the capacitance is decreased as the dielectric film thickness is increased, as expected. The ultrathin gate dielectric leads to an increase of the gate capacitance, thereby enhancing the performance of the device at the same gate bias. All the measurements were performed in an air environment.

**
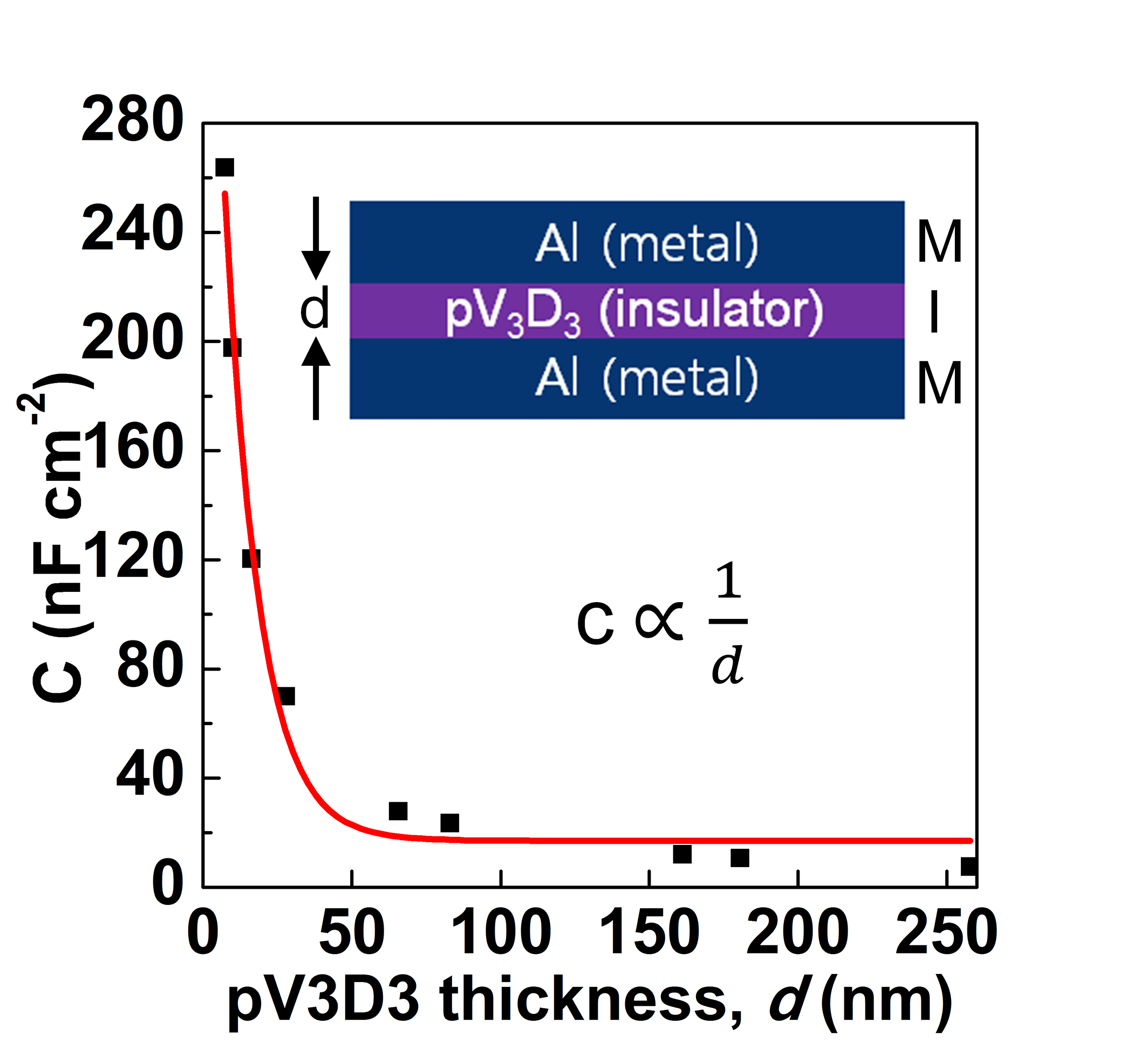
**

**Figure S2. Capacitance versus thickness of pV3D3 dielectric layer is analyzed for Al/pV3D3/Al (MIM) capacitor (top left).**

**Component analysis of the fabricated devices**

Figure S3 shows the results of the component analysis of the fabricated device using X-ray photoelectron spectroscopy (XPS) and energy-dispersive X-ray spectroscopy (EDS) mapping. The XPS spectrum confirmed the absence of impurities, which was expected because the iCVD process is solvent-free.


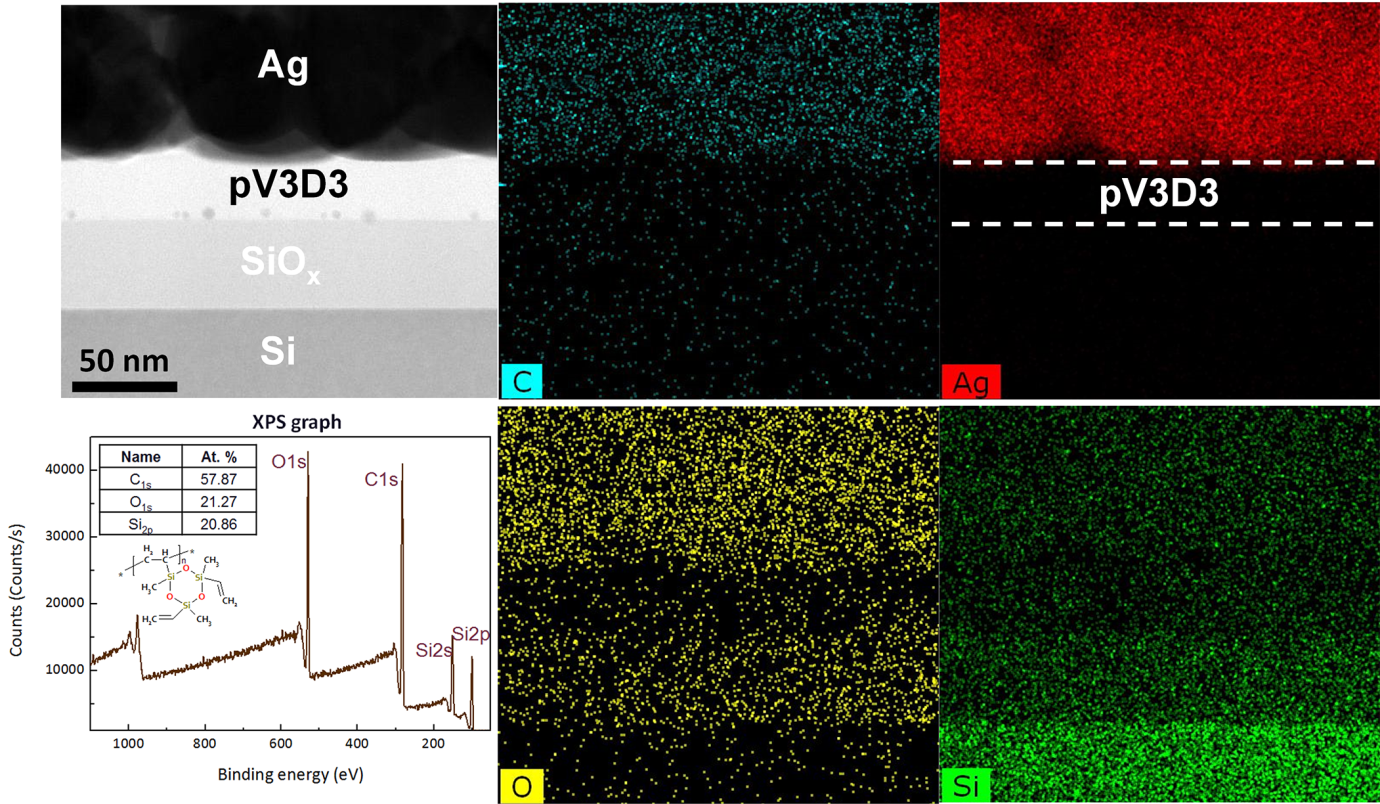


**Figure S3.** Cross-section TEM image of the fabricated device, EDS mapping analysis and high-resolution XPS spectra of the pV3D3 layer.

**Transfer characteristics of the CNT-TFTs with organic gate dielectrics**

Figure S4 shows the structures and the transfer characteristics of the CNT-TFTs with organic gate dielectrics. The transfer characteristics with the pV3D3 gate dielectric are obviously better than PMMA gate dielectric. Moreover, it should be noted that the ultra-thin (< 40 nm) gate dielectric possible in iCVD technique can contribute to the achievement of the abrupt switching characteristic via the improved gate controllability.

**
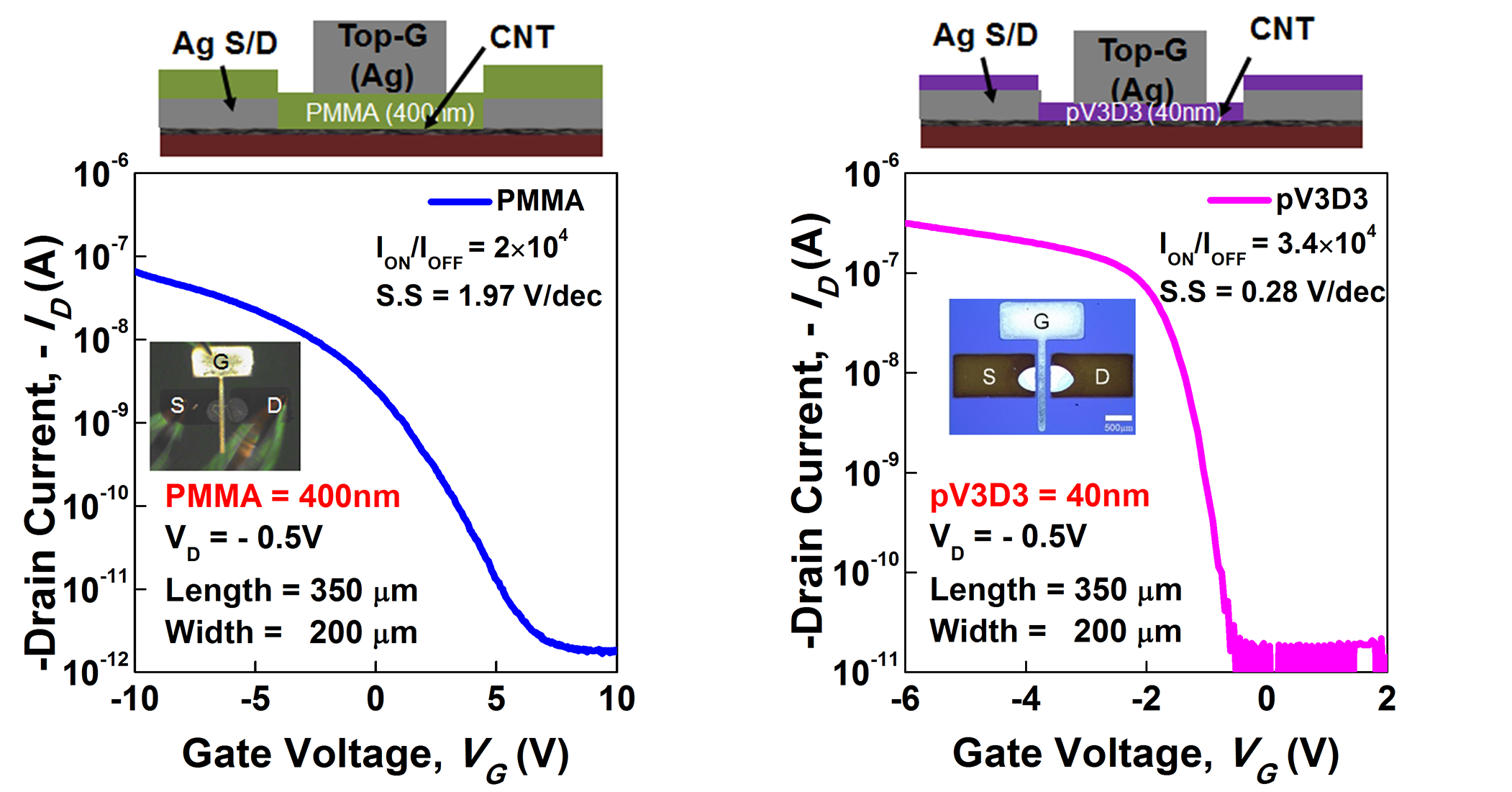
**

**Figure S4.** Comparison of transfer characteristics of the tog-gate CNT-TFTs with the PMMA and pV3D3 gate dielectric layer.

**Mechanical bending tests conducted on the flexible printed top-gate CNT-TFTs**

Figure S5 presents the results of repetitive bending tests conducted on the flexible printed top-gate CNT-TFTs with the pV3D3 gate dielectric. The transfer characteristics of the devices exhibited only minimal variations when bent down to a curvature radius as small as 2 mm and remained essentially unchanged throughout the process of up to 10^3^ bending cycles, as shown in Figures S5 (a) and 5(b). The electrical parameters such as *V_th_*, *I_ON_*/I_OFF_, and the mobility exhibited negligible variations from their initial values after the bending tests. In general, the bottleneck in the development of flexible integrated circuits is mainly caused by the fragile inorganic gate dielectric, which cracks much more easily relative to the metal thin film and CNT network upon aggressive bending. In this work, however, the bottleneck was found to be the printed Ag layers, which constitute the source, drain, and gate electrode, rather than the pV3D3 gate dielectric.^1^ As shown in Figures S5 (b) and 5(c), irreversible damage in the Ag layer caused a gradual increase in electrode resistance, which was responsible for the decreased on-state current and the mobility. Nevertheless, the experimental results revealed that the proposed CNT-TFTs can be applicable to general flexible devices. ^1-3^


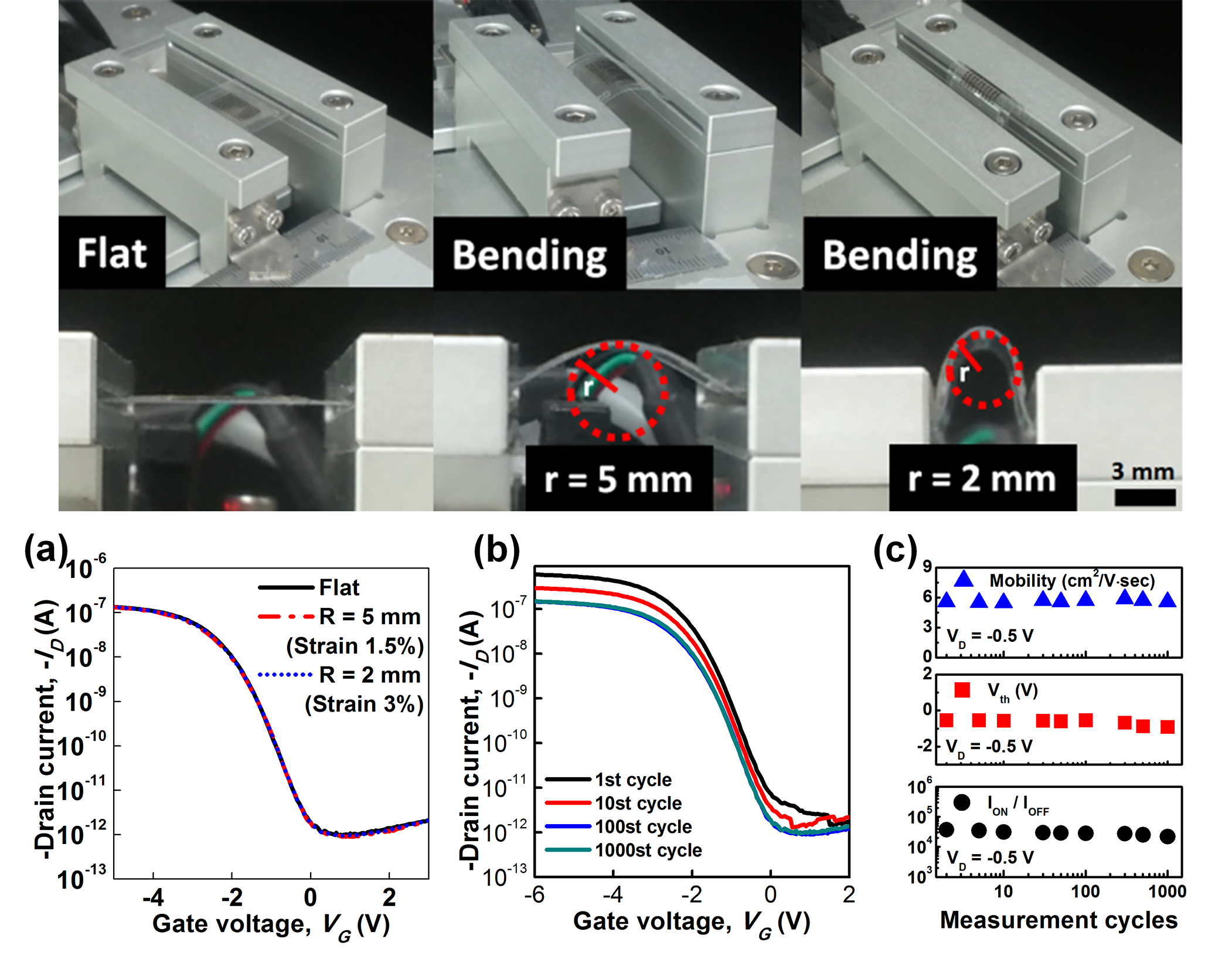
 **Figure S5.**Mechanical flexibility of the printed top-gate CNT-TFTs on a PES substrate. (a) Transfer I_D_ vs. V_G_ transfer characteristics of a representative device under several bending conditions measured at V_D_ - 0.5 V. (b) Transfer characteristics measured at V_D_ of -0.5 V as a function of the bending cycle. (c) V_th_, I_ON_/I_OFF_, and mobility versus the number of bending cycles.

**References**

1. Cai, L., Zhang, S., Miao, J., Yu, Z. & Wang, C. Fully Printed Foldable Integrated Logic Gates with Tunable Performance Using Semiconducting Carbon Nanotubes. *Advanced Functional Materials* **25,** 5698–5705 (2015).
2. Cao, X. *et al.* Screen Printing as a Scalable and Low-Cost Approach for Rigid and Flexible Thin-Film Transistors Using Separated Carbon Nanotubes. *ACS Nano* **8,** 12769–12776 (2014).
3. Moon, H. *et al.* Synthesis of ultrathin polymer insulating layers by initiated chemical vapour deposition for low-power soft electronics. *Nature Materials* **14,** 628–635 (2015).
